# What Is the Impact of Glyphosate on the Thyroid? An Updated Review

**DOI:** 10.3390/biomedicines13102402

**Published:** 2025-09-30

**Authors:** Lomesh Choudhary, Mathilda Monaghan, Rebecca Schweppe, Aime T. Franco, Whitney Goldner, Maaike van Gerwen

**Affiliations:** 1 Michael G. DeGroote School of Medicine, McMaster University, Hamilton, ON L8P 1A2, Canada; lomesh.choudhary@medportal.ca; 2 Department of Otolaryngology-Head and Neck Surgery, Icahn School of Medicine at Mount Sinai, New York, NY 10029, USA; mathilda.monaghan@mountsinai.org; 3 Division of Endocrinology, Metabolism, and Diabetes, Department of Medicine, University of Colorado School of Medicine, Anschutz Medical Campus, Aurora, CO 80045, USA; rebecca.schweppe@cuanschutz.edu (R.S.); whitney.goldner@cuanschutz.edu (W.G.); 4 Department of Pediatrics, Children’s Hospital of Philadelphia, Philadelphia, PA 19104, USA; francoa1@chop.edu; 5 Institute for Translational Epidemiology, Icahn School of Medicine at Mount Sinai, New York, NY 10029, USA

**Keywords:** glyphosate, herbicides, thyroid, endocrine disrupting chemicals, pesticide, TSH, thyroxine

## Abstract

**Background/Objectives**: Thyroid dysfunction (hypo- and hyperthyroidism) and cancer incidence have increased over the past decades, possibly linked to environmental contributions from endocrine disrupting chemicals (EDCs). Glyphosate is one of the most widely used herbicides globally and has endocrine-disruptive properties. Because of the sensitivity of the thyroid gland to endocrine disruption and the increased glyphosate exposure worldwide, this comprehensive review aimed to summarize studies investigating the link between glyphosate/glyphosate-based herbicides (GBHs) and thyroid dysfunction in human, animal, and in vitro studies. **Methods**: PubMed, Scopus, and Embase were used to search for original studies assessing glyphosate or GBH exposure and thyroid-related outcomes through December 2024. Data were extracted on study design, population or model, exposure, and thyroid outcomes. A total of 28 studies, including 9 human, 3 in vitro, and 16 animal studies were included. **Results**: Human studies showed mixed findings with some suggesting associations between glyphosate exposure and altered thyroid hormone levels, while others found no significant effects. Animal studies, particularly in rodents and amphibians, showed thyroid hormone disruption and altered gene expression, especially after perinatal or developmental exposure. In vitro studies reported changes in thyroid-related gene transcription and cell viability, however at concentrations exceeding those seen in humans. **Conclusions**: While there is some evidence that glyphosate may disrupt thyroid function, differences in study populations, exposure assessment methods, species models, and exposure doses complicated the comparison and summarization of the results. Further mechanistic and longitudinal studies are needed to clarify the thyroid-specific risks of glyphosate exposure.

## 1. Introduction

Over the past several decades, the incidence of thyroid dysfunction (hypo- and hyperthyroidism) and thyroid cancer has been rising in the United States [1,2]. There has been growing attention towards environmental risk factors, including endocrine disrupting chemicals (EDCs), as potential contributors to thyroid dysfunction [3,4]. Glyphosate—also known as N-(phosphonomethyl)glycine—is the most widely used herbicide worldwide. Glyphosate is commonly sold under the brand name Roundup^®^ [5]. It is a broad-spectrum, non-selective herbicide that has been in use since 1974 and is used in agricultural, residential, and industrial settings [6]. In the United States alone, glyphosate use in agriculture increased more than 14-fold over 20 years, reaching approximately 825,000 tons in 2014 [7]. Moreover, glyphosate exposure among Americans increased more than five-fold from 1996 to 2017, raising concerns about its potential health impacts [8].

Recent research has identified glyphosate and glyphosate-based herbicides (GBHs) as potential endocrine disruptors, capable of interfering with hormone signaling pathways in various human cell lines [9,10], and proposed mechanisms on how glyphosates disrupt thyroid hormone functioning [11]. Given the thyroid’s sensitivity to endocrine disruption and the widespread and increasing usage of glyphosate, it is important to evaluate all evidence linking glyphosate to thyroid outcomes. Therefore, this comprehensive review aimed to synthesize findings from human, animal, and in vitro studies published to date investigating the impact of glyphosate exposure on the thyroid.

## 2. Methods

**Figure 1:**
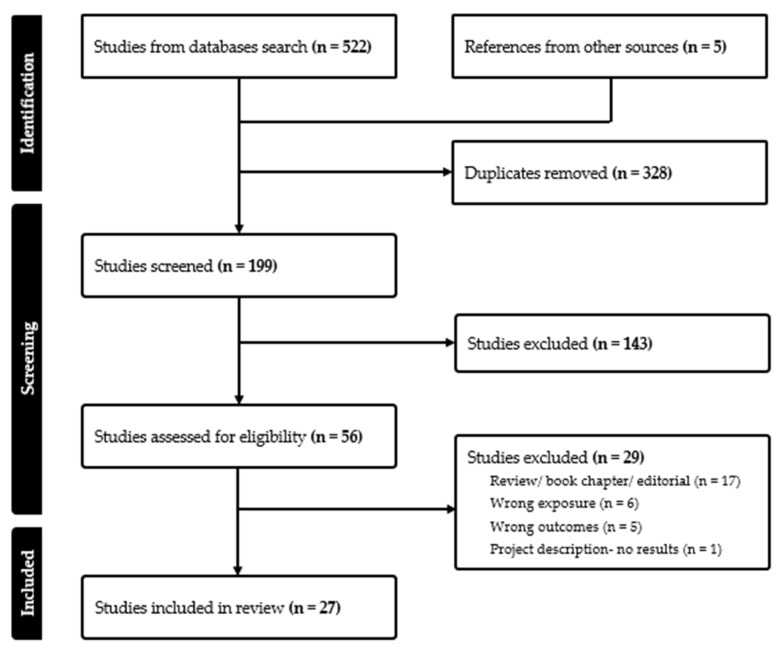
PRSIMA flow diagram of the studies selected for the review.

Original studies reporting on the impact of glyphosate exposure on thyroid-related outcomes were identified in the National Library of Medicine and National Institutes of Health PubMed, Scopus, and Ovid (Embase) databases. The search was conducted from inception to December 2024. To identify relevant publications, the following keyword search terms were used in various combinations: “glyphosate” or “N-(Phosphonomethyl)glycine)” or “(N-Phosphonomethyl-glycine)” or “Roundup^®^” or “Glyphosphate” or “N-Phosphonomethylglycine” or “N-Phosphomethylglycine” or “Glyfos” or “yerbimat” or “gliphosate” or “AMPA” or “Aminomethylphosphonic acid” and “thyroid”. Moreover, the reference lists from the retrieved articles and relevant reviews were reviewed for additional studies. Retrieved articles were imported and deduplicated in Covidence review software (Veritas Health Innovation, Melbourne, Australia) [12]. Titles and abstracts were reviewed for eligibility using the following eligibility criteria: (1) glyphosate or GBH exposure, (2) thyroid outcome, (3) human, animal, or in vitro studies. Reviews, systematic reviews, and meta-analyses were excluded but were reviewed for additional eligible studies. Data were extracted for the following variables from the included studies: primary author name, year of publication, country, study design, study population, experimental model, exposure, and measures of association (e.g., odds ratios (OR), hazard ratios (HR), and 95% confidence intervals (CI)). Measures of association were all presented as adjusted unless otherwise specified. We identified nine eligible studies on human effects, three in vitro studies, 15 animal studies (4 in tadpoles, 8 in rodents, and 3 in other animals) (Figure 1). Data are presented in a descriptive manner.

## 3. Results

### 3.1. Human Studies

In total, nine human studies were included [13,14,15,16,17,18,19,20,21], assessing the association between glyphosate exposure and various thyroid outcomes, such as alterations in thyroid hormone levels, development of hypothyroidism, and thyroid cancer. There were four prospective cohort studies [15,16,18,20], four retrospective case–control or cohort studies [14,17,19,21], and one cross-sectional study [13]. All studies were in an agricultural setting. Two studies included the female spouses of the licensed pesticide applicators [15,18], whereas the remaining seven studies assessed male applicators, farmers, or pesticide sprayers directly involved in glyphosate use [13,14,16,17,19,20,21]. Six studies were conducted in the United States [14,15,17,18,19,20], including four using the Agricultural Health Study (AHS) [15,17,18,20], two in Thailand [13,16], and one in Brazil [21] (Table 1). It should be noted that four studies used the same Agricultural Health Study data, and hence the results are similar amongst the studies [15,17,18,20].

#### 3.1.1. Thyroid Hormone Disruption

Four studies investigated associations between glyphosate exposure and alterations in thyroid hormone levels [13,16,19,21]. Kongtip et al. (2019) conducted a cross-sectional study in Thailand with 417 participants and found that increased glyphosate exposure was positively associated with slight elevations in serum T3 (triiodothyronine) and T4 (thyroxine) levels, with the strongest effect seen for total T4 (β = 1.007, 95% CI: 1.001–1.014) [13]. In a prospective study, Kongtip et al. (2021) found a significant increase in T4 levels observed with a mean change of 25.5 ng/dL (*p* = 0.045) when comparing the day before and the day after exposure in 48 pesticide sprayers [16].

A retrospective cohort study of Brazilian farm residents (Santos et al., 2019) observed a non-significant 22% decrease in TSH (thyroid-stimulating hormone) levels and modest but non-significant reductions in free and total T3 and T4 levels in individuals recently exposed to glyphosate when compared to individuals not exposed in the previous 7 days [21].

Lerro et al. (2018) assessed glyphosate exposure in male pesticide applicators and found no significant changes in TSH, T3, or T4 hormone levels. However, there was a slight trend suggesting that TSH levels might increase with higher exposure, which was close to being statistically significant (*p*-trend = 0.05) [19].

#### 3.1.2. Hypothyroidism

Five studies examined the potential association between glyphosate exposure and the development of hypothyroidism [15,17,18,19,20]. Shrestha et al. (2018) found that licensed pesticide applicators with a history of glyphosate use had a significantly increased risk of hypothyroidism (HR: 1.28; 95% CI: 1.07–1.52). There was a greater risk at moderate exposure levels (HR: 1.38; 95% CI: 1.12–1.69) and decreased risk at the highest exposure category, suggesting a possible non-linear dose–response relationship [20]. A separate study by Shrestha et al. (2018) examined female spouses of applicators and found no statistically significant association between glyphosate use and incident hypothyroidism (HR: 1.07; 95% CI: 0.95–1.20) or hyperthyroidism (HR: 0.90; 95% CI: 0.73–1.11). This suggests possible sex-, population-, or exposure-based differences [18].

Goldner et al. (2010) also evaluated hypothyroidism in a large cohort of 16,529 female spouses of pesticide applicators from the AHS and reported no significant association between glyphosate exposure and hypothyroidism (OR: 1.00; 95% CI: 0.91–1.20) [15]. Similarly, Lerro et al. (2018) found no significant association between glyphosate exposure and subclinical hypothyroidism among male pesticide applicators for 20–315 intensity-weighted days of glyphosate exposure (OR: 1.28; 95% CI: 0.71–2.32) and over 2622 intensity-weighted days (OR: 0.95; 95% CI: 0.51–1.77). There was no dose–response relationship (*p*-trend = 0.70) [19]. Finally, Goldner et al. (2013) explored thyroid disease among private male pesticide applicators and reported no increased odds of hypothyroidism among glyphosate users (OR; 1.18; 95% CI: 0.94–1.49) [17].

#### 3.1.3. Hyperthyroidism

Two studies investigated the association between glyphosate exposure and hyperthyroidism, though none reported statistically significant results. Shrestha et al. (2018) examined female spouses of licensed pesticide applicators and found no association between glyphosate use and incident hyperthyroidism (HR: 0.90; 95% CI: 0.73–1.11), after adjustment for potential confounders such as education, smoking, and correlated pesticide exposures [18]. Goldner et al. (2010) similarly assessed glyphosate exposure in a large cohort of female spouses and found no significant effect (OR: 0.98; 95% CI: 0.78–1.20) on hyperthyroidism [17].

#### 3.1.4. Thyroid Cancer

The potential link between glyphosate and thyroid cancer was only investigated by Omidakhsh et al. (2022). They reported a statistically significant association between glyphosate exposure and primary thyroid cancer of all subtypes (OR: 1.33; 95% CI: 1.12–1.58) in cases from the California Cancer Registry (1999–2012). Stratification by disease stage revealed elevated odds for both distant/regional (OR: 1.37; 95% CI: 1.08–1.73) and localized (OR: 1.26; 95% CI: 1.04–1.52) disease. However, these associations did not remain significant after adjusting for exposure to paraquat, a correlated pesticide [14].

### 3.2. In Vitro Studies

There were three in vitro studies that assessed the effects of glyphosate exposure on thyroid cells, using different cell lines and exposure durations [22,23,24]. The studies mainly focused on the impact of glyphosate and Roundup Original on thyroid-related gene expression and cell viability (Table 2).

Dal’ Bó et al. (2019) also evaluated the cytotoxicity and proliferative effects of Roundup Original on Nthy-ori 3–1 and TPC−1 cells. The Nthy-ori 3–1 line is a normal human thyroid follicular epithelial cell line that expresses thyroid-specific antigens such as TPO, Tg, NIS, and TSHR. In contrast, TPC−1 cells are derived from papillary thyroid carcinoma and represent a malignant model with different expression patterns of thyroid-related proteins. The study found that exposure to various concentrations of Roundup (6.5 µg/L to 6500 µg/L) resulted in varying levels of cell death, with the highest concentrations causing up to 53% and 57% death at 24 h. However, at 48 h, the cell viability remained above 79% for all the tested concentrations, with no significant differences in viability across concentrations. Moreover, the cell proliferation was also enhanced at lower concentrations, with Nthy-ori 3–1 cells exhibiting a 321% increase in proliferation at 6.5 µg/L after 48 h. TPC−1 cells showed a 208% increase in proliferation at the same concentration [22].

Ward et al. (2022) observed the effects of Roundup Original on the two thyroid-derived cell lines, Nthy-ori 3–1 (normal follicular cells) and TPC−1 (papillary carcinoma cells), over 24 and 48 h. Exposure to 160 µg/L and 830 µg/L concentrations of Roundup resulted in cell death in both cell lines, with the highest levels of death observed at 160 µg/L (52% and 58% for Nthy-ori 3–1 and TPC−1, respectively, at 24 h). After 48 h, the death or toxicity of the cell lines decreased but remained notable at 160 µg/L (19% and 29% for Nthy-ori 3–1 and TPC−1). In contrast, exposure to a lower concentration of 6.5 µg/L for 24 h induced cell proliferation, with cell viability increasing to 113% and 118% for Nthy-ori 3–1 and TPC−1 cells, respectively. This proliferative effect also occurred at 48 h, as measured by the CCK−8 assay [23].

Coperchini et al. (2023) investigated the impact of glyphosate exposure on thyroid-related gene expression in adherent−2D and spheroid−3D FRTL−5 cell models, which are cell lines originally established from normal rat thyroid glands. Exposure to 0.5 mM glyphosate for 24 h resulted in significant changes in the mRNA levels of several thyroid-related genes in both cell models. In the adherent−2D model, the mRNA levels of the NIS (sodium/iodide symporter), Pax8 thyroid transcription factor, TG (thyroglobulin), TSHR (thyroid-stimulating hormone receptor), and TTF−1 (thyroid transcription factor 1) were significantly upregulated. Similarly, in the spheroid−3D model, TG and TPO were significantly upregulated by 3.82- and 4.27-fold, respectively, while TSHR saw a dramatic increase of 14.76-fold (*p* < 0.05). These results suggest that glyphosate exposure can induce thyroid-related gene expression changes, which may have an impact on thyroid function [24].

### 3.3. Tadpole Studies

Four studies investigated the effects of glyphosate exposure on tadpoles focusing on thyroid hormone levels and gene expression across various developmental stages [25,26,27,28]. The four studies used different concentrations of glyphosate, and the exposure duration ranged from two weeks to several months (Table 3).

Howe et al. (2004) examined the relative expression levels of the thyroid hormone receptor *β* (*TRβ*) in the tails of North American amphibian species at Gosner stage 25 and 42. They did not find any significant differences in the mRNA levels between the control, 0.6 mg FAE/L glyphosate, and 1.8 mg FAE/L glyphosate groups at both Gosner stages [27].

Navarro-Martín et al. (2014) studied how VisionMax^®^ (glyphosate-based herbicide) exposure affected *Lithobates sylvaticus* tadpoles, using three groups, a control group, 0.021 mg a.e./L (acid equivalents per liter) exposure, and 2.9 mg a.e./L exposure. Herbicide exposure led to significant changes in thyroid-related gene expression, particularly in *trβ* (thyroid hormone receptor beta) and *dio2* (deiodinase type II). *Trβ* mRNA levels showed a dose-dependent increase, with a significant increase at 2.9 mg/L. Similarly, *dio2* expression was significantly altered at different Gosner stages, with an increase observed at GS35 and GS42 (F(4,69) = 16.163, *p* < 0.001). There were no significant changes observed in *dio3* expression across the different exposure concentrations [29].

Lajmanovich et al. (2019) assessed the acute and chronic toxicity of glyphosate-based herbicides (GHB) on *Rhinella arenarum* tadpoles. Acute (48 h) and chronic (22 days) exposure to 1.25 mg/L of glyphosate for 48 h resulted in non-significant changes in T3 and T4 levels [25].

Cuzziol Boccioni et al. (2021) assessed the effects of glyphosate exposure at two concentrations, 1.25 mg/L and 2.5 mg/L, on *Rhinella arenarum* tadpoles. They did not find any significant changes in thyroid hormone levels at the lower concentration (1.25 mg/L). However, when the tadpoles were exposed to 2.5 mg/L glyphosate, it resulted in a significant decrease in T4 levels (mean T4: 1.22 ± 0.1 ng/g, *p* > 0.005) compared to the control group (mean T4: 2.4 ± 0.75 ng/g) [26].

### 3.4. Rodent Studies

Eight studies assessed the effects of glyphosate exposure on thyroid function in rats and mice, focusing on hormone levels and gene expression [28,30,31,32,33,34,35,36]. These studies varied in exposed herbicide, exposure routes, durations, and doses of glyphosate. Most studies were performed in rats, with some including mice models. Studies investigated acute and chronic exposure to glyphosates (Table 4).

de Souza et al. (2017) exposed pregnant female Wistar rats to glyphosate by gavage at doses of 5 mg/kg/day and 50 mg/kg/day. The offspring were then analyzed for thyroid function. They found a significant reduction in TSH level in the higher dose (50 mg/kg/day) group, with TSH levels being 507.7 ± 91.49 ng/dL, significantly lower when compared to the control group (962.5 ± 152.1 ng/dL). However, T3 and T4 levels did not show any significant changes across the different exposure groups [30].

Hamdaoui et al. (2020) observed the effects of the herbicide Kalach 360 SL (a glyphosate formulation) on female Wistar rats exposed for 60 days at doses of 126 mg/kg and 315 mg/kg and found that glyphosate exposure resulted in a significant reduction in plasma T3 and T4 levels, with T3 levels dropping to 2.89 ± 1.27 pmol/L for the 126 mg/kg group and 2.35 ± 0.53 pmol/L for the 315 mg/kg group when compared to the control group (4.82 ± 0.76 pmol/L). Similarly, T4 levels decreased significantly, with the 315 mg/kg group showing a decrease to 13.26 ± 4.23 pmol/L from 25.83 ± 2.2 pmol/L, which was seen in the control group. Plasma TSH was increased, with the control group having a TSH of 0.457 +/− 0.123 pmol/L, the 126 mg/kg group having a TSH of 0.733 +/− 0.6 (*p* < 0.01), and the 315 mg/kg group having a TSH of 0.917 +/− 0.083 (*p* < 0.001) [32].

Costa Reis et al. (2021) exposed pregnant Wistar rats to glyphosate via gavage at doses of 5 mg/kg and 50 mg/kg. They measured gene expression in the thyroid gland and pituitary gland at postnatal days (PND) 60 and 90. Thyroid hormone receptor alpha 1 (*Thrα1*) mRNA levels were significantly elevated at the higher exposure dose (50 mg/kg: 1.513 ± 0.223), compared to controls (0.961 ± 0.272). The *Thrβ1* gene expression was significantly reduced at the 5 mg/kg dose (0.591 ± 0.343) compared to controls (1.147 ± 0.647), but there was no significant difference at the higher dose (0.957 ± 0.308). Similarly, *Dio3* gene expression was significantly decreased at the 5 mg/kg dose (0.534 ± 0.173). In contrast, the expression of *Mct8*, a thyroid hormone transporter, was significantly elevated at the 5 mg/kg dose (2.353 ± 0.482), indicating possible alterations in thyroid hormone transport. Other genes related to thyroid hormone synthesis and metabolism, such as *Dio2* and *Deiodinase 1*, did not show significant changes across the exposure groups [33].Docea et al. (2023) examined the effects of glyphosate exposure on thyroid function in female Wistar rats and found a significant reduction in total T3 and total T4 levels at 0.5 mg/kg bw/day. In the 0.5 mg/kg bw/day group, the total T3 levels were significantly reduced to 8.95 ± 0.59 ng/mL and the total T4 levels were significantly increased to 19.97 ± 1.73 nmol/L when compared to the control group (T3: 39.94 ± 1.11 ng/mL; T4: 13.30 ± 0.56 nmol/L). However, in the 50 mg/kg bw/day glyphosate group, the total T3 levels were significantly increased to 46.15 ± 2.83 ng/mL and the total T4 levels were significantly increased to 31.99 ± 1.17 nmol/L when compared to the control group (T3: 39.94 ± 1.11 ng/mL; T4: 13.30 ± 0.56 nmol/L). The TSH levels did not show significant changes for the 50 mg/kg bw/day group, but TSH was significantly increased for the 0.5 mg/kg bw/day group [35].

Oliveira et al. (2023) examined the effects of glyphosate exposure on male Wistar rats when administered 0.5 and 5 mg/kg/day via gavage for 37 days. They found that the TSH levels were significantly elevated at the higher dose (1.85 ± 0.3 ng/mL) compared to the control group (1.00 ± 0.14 ng/mL) at postnatal day (PND) 60, while no changes were found for T3 and T4 levels [36].

Manservisi et al. (2019) assessed the effects of glyphosate and Roundup Bioflow exposure on thyroid function in Sprague Dawley rats. At 6 weeks of exposure, glyphosate significantly increased the TSH levels in male rats (8.17 ± 1.58 ng/mL) when compared to the control (4.23 ± 0.76 ng/mL). A similar effect was observed in males after 13 weeks, with the TSH levels increasing from 1.89 ± 0.20 ng/mL in the control to 2.53 ± 0.25 ng/mL in the glyphosate-exposed group; however, this was not significant. The TSH levels showed no significant change in female rats [31].

Elkattan et al. (2024) exposed adult male albino rats to 1 mL of a 24% glyphosate solution for three weeks and found a significant decrease in free T3 and T4 levels, with free T3 levels being significantly lower (*p* < 0.05) in the exposure group (3.02 +/− 0.215) versus the control group (5.059 +/− 0.235). The total T3 levels were also significantly lower (*p* < 0.05) when compared to the controls in week 1 (93.9 +/− 13.1 in the exposed group and 126.9 +/− 7.2 in the control group) and week 2 (80.56 +/− 3.88 in the exposed group and 125.3 +/− 8.9 in the control group). Furthermore, free and total T4 levels were significantly decreased when compared to the control group for all three weeks. These changes were accompanied by an increase in TSH levels from 0.0144 ± 0.0098 μU/mL in the control group to 0.199 ± 0.0004 μU/mL (*p* < 0.05) by week 3 [28].

Zhang et al. (2021) conducted a study on female Kunming mice exposed to glyphosate at 250 and 500 mg/kg for seven days. When compared to the control groups, TRH levels were significantly elevated in the 250 mg/kg and 500 mg/kg glyphosate groups, while TSH, T4, and T3 levels were all significantly reduced in both exposure groups. Moreover, glyphosate resulted in hypothalamic–pituitary–thyroid (HPT) axis gene expression, including *dio2* and *mct8* mRNA level reductions for the low- and high-dose exposure groups when compared to the control in the hypothalamus, and *dio2*, *mct8*, and *Thrh* mRNA levels elevations in the low- and high-dose exposure groups when compared to the control in the pituitary gland [34].

### 3.5. Other Animal Model Studies

There were three studies that used other animal models to explore the thyroid-disrupting effects of glyphosate exposure, such as amphibians, zebrafish, and rabbits, using various glyphosate formulations and doses to assess changes in thyroid hormone levels and thyroid gland function [37,38,39] (Table 5).

## 4. Discussion

Liu et al. (2022) investigated the effects of 0.7 mg/L to 35 mg/L of glyphosate on thyroid function in zebrafish larvae (Danio rerio). Exposure to glyphosate led to significant changes in the ratio of T3 to T4 at 120 h post-fertilization, with the highest concentration (35 mg/L) resulting in a decreased ratio of 0.0148 ± 0.0009 compared to the control group (0.0209 ± 0.0013). The study also found significant decreases in T3 levels at the highest glyphosate concentration (7.61 ± 0.37 pmol/L) when compared to the control (9.31 ± 0.65 pmol/L). T4 levels were also altered, with the highest concentration (35 mg/L) showing a significant increase in T4 (513.6 ± 25.4 pmol/L) when compared to the control group (441.7 ± 7.9 pmol/L) [37].

Vardakas et al. (2022) found no significant differences in thyroid gland antioxidant capacity or glutathione levels after exposing rabbits to glyphosate and Roundup. However, there was a significant reduction in catalase activity in the thyroid gland of the Roundup-exposed group (48.78 ± 6.61 U/mg protein) when compared to the control group (67.84 ± 7.36 U/mg protein) [38].

Killian et al. (2023) found that exposure to glyphosate at concentrations ranging from 0.05 mg/L to 20 mg/L led to significant alterations in the expression of *TRα* and *TRβ* thyroid hormone receptors in *Oryzias latipes* (Japanese medaka) larvae. At 20 mg/L, *TRα* expression was significantly reduced (0.274 ± 0.182) compared to controls (1.523 ± 0.746). Similarly, *TRβ* expression was significantly reduced at 20 mg/L (0.264 ± 0.132) when compared to the control group (1.22 ± 0.437) [39].

This review provides an in-depth summary of human, in vitro, and animal studies investigating the effects of glyphosate exposure on thyroid function. Although there are indications suggesting a potential link between glyphosate exposure and changes in thyroid hormone levels, thyroid-related gene expression, and thyroid gland function, the evidence remains mixed and limited in strength due to the variability in study design, exposure measurements, and outcomes assessed.

Glyphosate is the most widely used herbicide worldwide and has endocrine-disruptive properties [6,9,10]. While several human studies confirmed that glyphosate exposure leads to disruption in thyroid hormone levels, findings have been inconsistent; this is potentially due to differences in study populations (applicators [13,14,16,19,21] versus spouses [15,17,18,20]), exposure assessment (self-reporting versus biomonitoring), co-exposures to other pesticides, and geographic location (e.g., US, Brazil, Thailand [13,16,21]). Similar issues explaining mixed results in animal and in vitro studies can be listed, such as different species (tadpoles [25,26,27,29], rodents [28,30,31,32,33,34,35,36], zebrafish [37], New Zealand rabbits [38], and Japanese medaka larvae [39]) and developmental stages (both gestational exposure and postnatal days [30,31,32,33,34,35] versus only postnatal exposure [36]), or concentrations of glyphosate (from 6.5 µg/L [22] to high exposure of 50 mg/kg/day [30]). This immediately highlights the difficulties that exist when investigating the effect of an exposure directly impacting the applicability of the results to the general population.

Although the exact mechanism of how glyphosate affects thyroid and endocrine functioning is unknown, the results bring light to a few hypotheses. In rodents, it is seen that glyphosate may decrease TSH, T3, and T4 levels by interfering with the hypothalamic–pituitary–thyroid (HPT) axis [28,34]. When this was examined in cell models, it was found that glyphosate modulates the gene transcription of thyroid-related genes, such as the genes coding for NIS, TG, TPO, TSHR, and transcription factors such as *TTF−1* and *Pax8* [24]. Furthermore, glyphosate has been shown to significantly upregulate thyroid-specific genes such as *TSHR*, *TG*, and *TPO* in both 2D and 3D FRTL−5 cell models, suggesting overstimulation of thyroid growth and function [24]. The effects can vary by species developmental stage. In embryo-larval zebrafish, 35 mg/L of glyphosate resulted in a significant decrease in T3 and significant increase in T4, suggesting that high doses may inhibit the conversion of T4 into the active T3 [37]. Similarly, perinatal and postnatal exposure in rodent models led to altered expression of thyroid hormone transporters and receptors [30,33]. The developmental sensitivity is important as thyroid hormones are regulators of cell differentiation, growth, and apoptosis during fetal and postnatal life [40]. Disruption during this window could lead to permanent changes in the architecture or function of the thyroid gland, possibly increasing the chance of neoplasm later in life. In comparison, glyphosate exposure in adult Wistar rats led to TSH elevation without consistent T3 and T4 changes, indicating a different compensatory response than that seen in developmental stages [36]. Finally, regardless of the concentration and cell type (normal thyroid follicular cells or cells from papillary thyroid cancer), glyphosate resulted in thyroid cell death; however there was no significant dose-dependent relationship observed [22,23]. It should be noted that a paradoxical effect was observed, where early time points showed increased cell death alongside increased proliferation. This pattern suggests that cells that survive the initial toxicity may undergo proliferative changes, contributing to the cellular transformation. More in vitro studies looking at long-term exposure are needed to assess the apparent paradoxical response [22,23]. It may also alter the gene transcription of thyroid hormone receptors and enzymes involved in synthesis and metabolism [30,41,42].

This review has several limitations. Due to the differences in study designs, sample sizes, methods of exposure, and outcome measurements, it is difficult to directly compare the results of the included studies. For the human studies, many of them relied on self-reported exposure, which has the potential for recall bias. The animal and in vitro cell studies may not accurately reflect real-world circumstances, as the methods and concentration of glyphosate exposure would not be seen in humans. Lastly, there is limited data for the long-term and low-dose effects of glyphosate exposure in human, in vitro, and animal studies as well as for potential effect of combined exposure with other herbicides.

## 5. Conclusions

In summary, glyphosate exposure has been linked to thyroid hormone alterations, gene expression changes, and thyroid dysfunction in animal and in vitro studies, but human evidence remains inconsistent. The differences in study design, exposure assessment, and species make direct comparisons complicated. Moving forward, longitudinal human studies are needed to clarify whether glyphosate poses a clinically significant risk for thyroid disease, particularly in low-dose, chronic exposure settings, which better represents real-world scenarios. These studies should also include autoimmune thyroid disease, pregnancy-related thyroid anomalies amid exposure, or neonatal impact as this information is currently lacking. Animal and in vitro studies should also employ concentrations and exposure durations that more accurately reflect this. Further research should look at the sex-specific effects and how glyphosate interacts with other herbicides to disrupt thyroid functioning. Since glyphosate is the most used herbicide in the world and thyroid hormones play an essential role in metabolism and development, further research is needed to assess the potential health outcomes to inform future policies regarding safe glyphosate usage.

## Figures and Tables

**Table 1:** Summary of human studies investigating the association between glyphosate exposure and thyroid disease.

Author (Year)	Study Design	Study Population (*n*)	Country	Number of Cases and Controls	Association with Thyroid Disease
Goldner et al. (2010) [15]	Prospective cohort study	Female spouses of licensed pesticide applicators (*n* = 16,529)	US	5071 exposed cases and 11458 control cases	Hyperthyroidism: OR 0.98 (95% CI: 0.78–1.2) Hypothyroidism: OR 1.00 (95% CI: 0.91–1.2) Other thyroid disease: OR 0.97 (95% CI: 0.81–1.2)
Goldner et al. (2013) [17]	Retrospective cohort study	Private male pesticide applicators (*n* = 22,246)	US (AHS); Iowa and North Carolina	919 exposed cases and 21,327 controls)	Hypothyroidism: 1.18 (95% CI 0.94–1.49)
Lerro et al. (2018) [19]	Retrospective cohort study	Male pesticide applicators (*n* = 679)	US (AHS); Iowa and North Carolina	552 total exposed, 127 total controls	Glyphosate exposure intensity-weighted days (0 days: reference): 20–315 days: Subclinical hypothyroidism: OR 1.28 (95% CI: 0.71–2.32). Natural log of hormones: TSH: beta: 1.02 (95% CI: 0.88–1.17), T4: beta: 1.01, (95% CI: 0.95–1.07), and T3: beta: 0.98, (95%CI: 0.92–1.04). >315–917 days: Subclinical hypothyroidism: OR 1.28 (95% CI: 0.6–1.93). Natural log of hormones: TSH: beta: 1.00 (95% CI: 0.87–1.15), T4: beta: 1.01 (95% CI: 0.95–1.07), and T3: beta: 1.01 (95% CI: 0.95 to 1.07). >907–2622 days: Subclinical hypothyroidism: OR: 0.95 (95% CI: 0.51–1.77). Natural log of hormones: TSH: beta: 1.00 (95% CI: 0.87–1.15), T4: beta: 1.01 (95% CI: 0.95–1.07), and T3: beta: 1.01 (95% CI: 0.95–1.07). >2622–113,400 days: Subclinical hypothyroidism: OR: 1.21 (95% CI: 0.66–2.24). Natural log of hormones: TSH: beta: 1.14 (95% CI: 0.99–1.33), T4: beta: 0.99 (95% CI: 0.94–1.05), and T3: beta: 0.97 (95% CI: 0.91–1.01). *p*-trend for subclinical hypothyroidism: 0.70 *p*-trend for TSH: 0.05. *p*-trend for T4: 0.68. *p*-trend for T3: 0.41
Shrestha et al. (2018) [20]	Prospective cohort study	Licensed pesticide applicators (*n* = 821)	US	663 exposed cases with hypothyroidism, 158 controls	Ever use of glyphosate and hypothyroidism risk: **1.28 (95% CI 1.07–1.52)** Intensity-weighted lifetime days of use of glyphosate and hypothyroidism risk: **>0–≤686 intensity-exposed days: HR: 1.27 (95% CI: 1.03–1.56)** **>686–≤2604 intensity-exposed days: HR: 1.38 (95% CI: 1.12–1.69)** >2604 intensity-exposed days: HR: 1.17 (95% CI: 0.94–1.45)
Shresta et al. (2018) [18]	Prospective cohort study	Female spouses of licensed pesticide applicators (*n* = 2087)	US	755 exposed cases (170 with hyperthyroidism and 585 with hypothyroidism), 1332 controls (345 with hyperthyroidism and 987 with hypothyroidism)	Ever use of pesticides and risk of incident hypothyroidism: HR: 1.05 (95% CI 0.94–1.16) when adjusted for education, state, and smoking, and HR: 1.07 (95% CI 0.95–1.20) when additionally adjusted for correlated pesticides, wherever applicable. Ever use of pesticides and risk of incident hyperthyroidism: HR: 0.93 (95% CI 0.77–1.12) when adjusted for education, state, and smoking, and HR: 0.90 (95% CI 0.73–1.11) when additionally adjusted for correlated pesticides.
Kongtip et al. (2019) [13]	Cross-sectional study	Farmers (*n* = 417)	Thailand	222 non-exposed participants (114 male, 108 female), 195 exposed participants (144 male, 51 female)	TSH: beta: 0.992 (95% CI 0.957–1.027) FT3: beta: 1.002 (95% CI 0.998–1.007) FT4: beta: 0.999 (95% CI 0.993–1.005) T3: beta: 1.006 (95% CI 0.999–1.012) **T4: beta: 1.007 (95% CI 1.001–1.014)**
Santos et al. (2019) [21]	Retrospective cohort study	Farm residents (*n* = 122)	Brazil	97 cases and 25 controls	Percent change in serum hormone levels with recent glyphosate exposure when comparing exposed to unexposed in the past 7 days: TSH: −22% (95% CI: −56 to 37); Free T4: −1% (95% CI: −10 to 8); Total T4: −7%(95% CI: −20 to 8); Free T3: −5% (95% CI: −13 to 5); Total T3: 0% (95% CI: −13 to 15)
Kongtip et al. (2021) [16]	Prospective cohort study	Pesticide sprayers (*n* = 48)	Thailand	48 exposed	Change in thyroid hormone levels as a function of changes in urinary glyphosate levels after herbicide spraying (both logged morning after spraying—logged morning before spraying (ΔLN)) ΔLNTSH (nIU/mL). Beta = 68.1. SE = 51.1. *p*-value = 0.183. ΔLNFT3 (pg/dL). Beta = 1.7. SE = 12.1. *p*-value = 0.888. ΔLNFT4 (pg/dL). Beta = 11.1. SE = 10.9. *p*-value = 0.311. ΔLNT3 (ng/dL). Beta = 11.7. SE = 25.1. *p*-value = 0.642. **ΔLNT4 (ng/dL). Beta = 25.5. SE = 12.1. *p*-value = 0.045.**
Omidakhsh et al. (2022) [14]	Retrospective case–control study	Residents of central California agricultural area (*n* = 3070)	US, central California	2067 cases and 1003 controls	**Primary thyroid cancer of all subtypes: 1.33 (95% CI: 1.12–1.58)** **Distant/regional thyroid cancer: OR 1.37 (95% CI: 95% 1.08, 1.73)** **Localized thyroid cancer: OR 1.26 (95% CI: 1.04, 1.52).** (Disease associations did not remain after adjustment for paraquat)

Bolded values are used to indicate statistical significance. OR = Odds Ratio; HR = Hazard Ratio; CI = Confidence Interval; AHS = Agricultural Health Study; TSH = Thyroid-Stimulating Hormone; T3 = Triiodothyronine; T4 = Thyroxine; FT3 = Free Triiodothyronine; FT4 = Free Thyroxine; β = Regression Coefficient.

**Table 2:** Summary of in vitro studies investigating the impact of glyphosate exposure on thyroid-related outcomes (gene expression/cell effects).

Author (Year)	Chemical Exposure	Experimental Model	Exposure Duration	Association with Thyroid Disease
Dal’ Bó et al. (2019) [22]	Roundup Original Dl	Nthy-or 3–1 (from thyroid normal follicular cells) TPC−1 (from papillary carcinoma)	24 and 48 h	Percent mortality of Nthy-ori 3–1 and TPC−1 cells measured by trypan blue exclusion test. 24 h: 6.5 µg/L Roundup: 49.5 +/− 1.5% for Nthy-ori 3–1. 54 +/− 9.5% for TPC−1. 65 µg/L Roundup: 38 +/− 3.7% for Nthy-ori 3–1. 63 +/− 8.9% for TPC−1. 160 µg/L Roundup: 43 +/− 9.2% for Nthy-ori 3–1. 50 +/− 7.5% for TPC−1. 830 µg/L Roundup: 35 +/− 8.4% for Nthy-ori 3–1. 58 +/− 3.7% for TPC−1. 6500 µg/L Roundup: 53 +/− 3% for Nthy-ori 3–1. 57 +/− 9.6% for TPC−1. 48 h: 6.5 µg/L Roundup: 26 +/− 12.6% for Nthy-ori 3–1. 22 +/− 6% for TPC−1. 65 µg/L Roundup: 34 +/− 5.7% for Nthy-ori 3–1. 31 +/− 4.8% for TPC−1. 160 µg/L Roundup: 33 +/− 15.1% for Nthy-ori 3–1. 33 +/− 4.9% for TPC−1. 830 µg/L Roundup: 18 +/− 3.8% for Nthy-ori 3–1. 37 +/− 14.7% for TPC−1. 6500 µg/L Roundup: 23 +/− 3.8% for Nthy-ori 3–1. 15 +/− 1.7% for TPC−1. Percent of viable cells after Roundup exposure measured by CCK−8 assay. 24 h. 6.5 µg/L Roundup: 113 +/− 13.44% for Nthy-ori 3–1. 105 +/− 15.3% for TPC−1. 65 µg/L Roundup: 104 +/− 7.55% for Nthy-ori 3–1. 94 +/− 6.6% for TPC−1. 160 µg/L Roundup: 87 +/− 3.6% for Nthy-ori 3–1. 88 +/− 6.6% for TPC−1. 830 µg/L Roundup: 87 + /− 5.36% for Nthy-ori 3–1. 97 +/− 11.1% for TPC−1. 6500 µg/L Roundup: 86 +/− 6.23% for Nthy-ori 3–1. 91 +/− 6.9% for TPC−1. 48 h. 6.5 µg/L Roundup: 101 +/− 1.2% for Nthy-ori 3–1. 79 +/− 24.8% for TPC−1. 65 µg/L Roundup: 102 +/− 1.73% for Nthy-ori 3–1. 79 +/− 16.2% for TPC−1. 160 µg/L Roundup: 84 +/− 4.18% for Nthy-ori 3–1. 100 +/− 7.83% for TPC−1. 830 µg/L Roundup: 91 +/− 4.48% for Nthy-ori 3–1. 82 +/− 12.3% for TPC−1. 6500 µg/L Roundup: 92 +/− 1.76% for Nthy-ori 3–1. 106 +/− 4.93% for TPC−1.
Ward et al. (2022) [23]	Roundup Original Dl	Thyroid-derived cell lines Nthy-or 3–1 (from thyroid normal follicular cells) and TPC−1 (from papillary carcinoma)	24 and 48 h	At 24 h, 160 µg/L resulted in 52% and 58% cell death in Nthy-ori 3–1 and TPC−1 cells, respectively. 830 µg/L resulted in 43% and 58% cell death in Nthy-ori 3–1 and TPC−1 cells, respectively. At 48 h, 160 µg/L resulted in 19% and 29% cell death in Nthy-ori 3–1 and TPC−1 cells, respectively. 830 µg/L resulted in 15% and 23% cell death in Nthy-ori 3–1 and TPC−1 cells, respectively. After 24 h of 6.5 µg/L exposure, cell viability increased to 113% in Nthy-ori 3–1 cells and 118% in TPC−1 cells. This proliferative effect persisted at 48 h as measured by the CCK−8 assay
Coperchini et al. (2023) [24]	Glyphosate	Adherent−2D and spheroid−3D models derived from the Fisher rat thyroid cell line−5 (FRTL−5) cell strain	24 h	Changes in the mRNA levels of thyroid-related genes after Adherent and spheroid FRTL−5 cells were exposed to 0.5 mM glyphosate: FRTL−5 Adherent−2D Model: **NIS** (sodium/iodide symporter)**: 1.85-fold change (95% CI: 1.13–3.17)** ***Thyroid transcription factor* gene *Pax8*: 2.34-fold change (95% CI: 1.77–6.53)** ***TG***(thyroglobulin)**: 2.49-fold change (95% CI: 1.58–3.36)** *TPO* (thyroid peroxidase): 1.85-fold change (95% CI: 0.94- 2.94) ***TSHR***(thyroid-stimulating hormone receptor)**: 2.75-fold change (95% CI: 1.51–8.34)** **TTF−1** (thyroid transcription factor 1)**: 3.55-fold change (95% CI: 1.92–4.11)** FRTL−5 Spheroid−3D Model: ***TG*: 3.82-fold change (95% CI: 2.31–5.60)** ***TPO*: 4.27-fold change (95% CI: 3.02–7.29)** ***TSHR*: 14.76-fold change (95% CI: 1.69–25.78)**

Bolded values are used to indicate statistical significance. Nthy-ori 3–1 = Normal thyroid follicular cell line; TPC−1 = Papillary thyroid carcinoma cell line; FRTL−5 = Fisher rat thyroid cell line; NIS = Sodium/Iodide Symporter; TG = Thyroglobulin; TPO = Thyroid Peroxidase; TSHR = Thyroid-Stimulating Hormone Receptor; TTF−1 = Thyroid Transcription Factor 1; Pax8 = Paired box gene 8; CCK−8 = Cell Counting Kit−8 viability assay; mRNA = Messenger RNA; µg/L = Micrograms per liter; mM = Millimolar.

**Table 3:** Summary of tadpole studies investigating the impact of glyphosate exposure on thyroid-related outcomes (hormone levels/gene expression).

Author (Year)	Chemical Exposure and Dose	Experimental Model and Exposure Route	Exposure Duration and Age at Experiment End	Association with Thyroid Disease
Howe et al. (2004) [27]	Glyphosate technical and Roundup Original; 0.6 and 1.8 mg FAE/L (formulation acid equivalents)	North American amphibian species (*Rana clamitans*,* R. pipiens*,* R. sylvatica*, and *Bufo americanus*); Immersion in aquaria filled with filtered river water and the test compounds added once a week in a static renewal system	Acute exposure had 24 and 96 h exposures; chronic exposure groups were exposed until 80% or more of the surviving tadpoles in each control group reached metamorphic climax (Gosner stage 42).	Relative expression levels (copy number/5 ng total RNA) of thyroid hormone receptor ß (*TRß*) mRNA in the tails of Gosner stage 25: Control: 901.8 +/− 34.9 0.6 mg FAE/L glyphosate: 913.5 +/− 87.2 1.8 mg FAE/L glyphosate: 1189.8 +/− 75.7 Gosner stage 42: Control: 10349 +/− 1327 0.6 mg FAE/L glyphosate: 0 1.8 mg FAE/L glyphosate: 8874.7 +/− 884.5
Navarro-Martína et al. (2014) [29]	VisionMax (glyphosate-based herbicide): 0.021 mg acid equivalents (a.e.)/L and 2.9 mg a.e./L.	Wood frog tadpoles (*Lithobates sylvaticus*); Water	Exposure started at GS 25; GS 42 at the end of the experiment.	Fold change of trß, *dio2*, *dio3* mRNA levels in the brain from GS30 to GS42: *trß*: **Increased *trß* expression across developmental stages (F(2,69) = 3.475, *p* = 0.037)** *dio2*: **Significant interactions between treatment and Gosner developmental stage were detected for *dio2* (F(4,69) = 16.163, *p* < 0.001).** *dio3*: Not significant by treatment (F(2) = 2.249, *p* = 0.118). Fold change of *trß*, *dio2*, *dio3* mRNA levels in the tails of tadpoles at GS30 to GS42 chronically exposed to VisionMax^®^ (VM^®^), determined by real-time RT-PCR. *trß*: **Significant treatment effect: (F(2,69) = 27.569, *p* < 0.001)** *dio2*: **Significant treatment and stage interaction: (F(4,69) = 11.157, *p* < 0.001)** *dio3*: Not significant by treatment (F(2) = 2.653, *p* = 0.079)
Lajmanovich et al. (2019) [25]	Glyphosate-based herbicide (GBH): Acute toxicity: 1.25 mg/L of GBH. Chronic toxicity: 1.25 mg/L of GBH	Tadpoles (*Rhinella arenarum*); Immersion in test solutions containing the GBH in glass flasks with dechlorinated tap water	Acute toxicity was exposed for 48 h, and chronic toxicity was exposed for 22 days; All tadpoles were at GS 26–30 at the start of the experiment	T3 levels in the 48 h toxicity group: Control: 1.714 +/− 0.187 ng/g GBH: 1.592 +/− 0.248 ng/g T3 levels in the 22 days toxicity group: Control: 1.149 +/− 0.232 ng/g GBH: 1.199 +/− 0.215 ng/g T4 levels in the 48 h toxicity group: Control: 6.441 +/− 1.299 ng/g GBH: 6.723 +/− 1.695 ng/g T4 levels in the 22 days toxicity group: Control: 3.842 +/− 1.808 ng/g GBH: 5.819 +/− 2.034 ng/g
Cuzziol Boccioni et al. (2021) [26]	Glyphosate: 1.25 mg/L (GHB1) and 2.5 mg/ L (GHB2)	Tadpoles (*Rhinella arenarum)*; Water	2 weeks GS (Gosner stage) 26 at the end of the experiment.	Control: mean T4: 2.4 +/− 0.75 ng/g GHB1: 2.66 +/− 0.36 ng/g **GHB2: mean T4: 1.22 +/− 0.1 ng/g (*p* > 0.005)**

Bolded values are used to indicate statistical significance. GS = Gosner Stage; TRβ = Thyroid Hormone Receptor Beta; Dio2 = Type II Deiodinase; Dio3 = Type III Deiodinase; mRNA = Messenger RNA; ng/g = Nanogram per gram body weight; a.e. = Acid Equivalents; FAE/L = Formulation Acid Equivalents per Liter.

**Table 4:** Summary of rodent studies investigating the impact of glyphosate exposure on thyroid-related outcomes (hormone levels/gene expression).

Author (Year)	Chemical Exposure and Dose	Experimental Model and Exposure Route	Age at the Beginning of the Exposure and Exposure Duration	Association with Thyroid Disease
de Souza et al. (2017) [30]	Roundup Transorb; 5 mg/kg/day and 50 mg/kg/day	Female pregnant Wistar rats were exposed, male offspring were analyzed; By Gavage	GD (gestational day) 18 at the beginning of the exposure; Exposure lasted until PND (postnatal day) 5	TSH (ng/dL): Control: 962.5 +/− 152.1 **5 mg/kg/day: 526.3 +/− 96.32 (*p* < 0.05)** **50 mg/kg/day: 507.7 +/− 91.49 (*p* < 0.05)** T3 (ng/dL): control: 58.63 +/− 4.590 5 mg/kg/day: 48.13 +/− 2.730 50 mg/kg/day: 52.36 +/− 2.123 T4 (ng/dL): control: 4.674 +/− 0.2476; 5 mg/kg/day: 4.335 +/− 0.2404; 50 mg/kg/day:4.801 +/− 0.1748
Manservisi et al. (2019) [31]	Glyphosate or Roundup Bioflow; Dose of 1.75 mg/kg bw/day	Sprague Dawley rats; Drinking water	GD 6 (in utero) up to PND 120; Exposed for 6 weeks and 13 weeks.	TSH (males; mean ± SEM): 6-week cohort: control 4.23 ± 0.76, **glyphosate 8.17 ± 1.58 (*p* < 0.05)**, Roundup 5.57 ± 0.31 13-week cohort: control 1.89 ± 0.20, glyphosate 2.53 ± 0.25, **Roundup 3.69 ± 0.42 (*p* < 0.01)** TSH (females; mean ± SEM): 6-week cohort: control 2.70 ± 1.13, glyphosate 3.02 ± 2.00, Roundup 3.04 ± 1.53 13-week cohort: control 1.29 ± 0.69, glyphosate 1.93 ± 0.89, Roundup 3.03 ± 2.22
Hamdaoui et al. (2020) [32]	Kalach 360 SL herbicide (KL); 126 mg/Kg and 315 mg/Kg dissolved in water	Female Wistar rats; Gavage, dissolved in water.	Rats were exposed for 60 days	Plasma-Free T3 Levels (pmol/L). control: 4.82 +/− 0.76 **126 mg/Kg: 2.89 +/− 1.27 (*p* < 0.01)** **315 mg/Kg: 2.35 +/− 0.53 (*p* < 0.001)** Plasma-Free T4 levels (pmol/L): control: 25.83 +/− 2.2 **126 mg/Kg: 16.85 +/− 2.61 (*p* < 0.05)** **315 mg/Kg: 13.26 +/− 4.23 (*p* < 0.01)** Plasma TSH levels (pmol/L) control 0.457: +/− 0.123 **126 mg/Kg: 0.733 +/− 0.6 (*p* < 0.01)** **315 mg/Kg: 0.917 +/− 0.083 (*p* < 0.001)**
Costa Reis et al. (2021) [33]	Roundup Transorb; 5 mg/kg/day and 50 mg/kg/day	Female pregnant Wistar rats were exposed, male offspring were analyzed; By Gavage	GD 18 to PND 5	Thyroid hormone receptor alpha 1 (*Thrα1*) mRNA levels: control 0.961 ± 0.272, 5 mg/kg 0.906 ± 0.238, **50 mg/kg 1.513 ± 0.223 (*p* < 0.05)** Receptor *Thrβ1* gene expression: control 1.147 ± 0.647, **5 mg/kg 0.591 ± 0.343 (*p* < 0.05)**, 50 mg/kg 0.957 ± 0.308 Receptor *Thrβ2* gene expression: control 1.116 ± 0.562, 5 mg/kg 0.698 ± 0.280, and 50 mg/kg 0.835 ± 0.209 Thyroid hormone transporter *Slco1c* (*Oatp1c1*) expression: control 1.302 ± 0.988, 5 mg/kg 1.386 ± 0.855, and 50 mg/kg 1.277 ± 0.950 Thyroid hormone transporter, *Mct8*, expression: control 1.114 ± 0.662, **5 mg/kg 2.353 ± 0.482 (*p* < 0.05)**, 50 mg/kg 1.909 ± 0.988 *Deiodinase 1* gene expression: control 1.335 ± 0.896, 5 mg/kg 1.257 ± 1.513, and 50 mg/kg 1.184 ± 0.850 *Deiodinases 2* (*Dio2*) gene expression: controls 1.027 ± 0.236, 5 mg/kg 0.968 ± 0.692, and 50 mg/kg 1.008 ± 0.606 *Dio3* gene expression: controls 1.554 ± 0.866, **5 mg/kg 0.534 ± 0.173 (*p* < 0.05)**, 50 mg/kg 0.891 ± 0.568
Zhang et al. (2021) [34]	Glyphosate; Low dose: 250 mg/kg High dose: 500 mg/kg	Female Kunming mice; Intragastric	4 weeks old at the beginning of exposure; 7 days of exposure	TRH (µ IU/mL): control: 7.8 +/− 1.92, **Low dose: 14.93 +/− 1.08 (*p* < 0.0001), High dose: 14.93 +/− 1.12 (*p* < 0.0001)** TSH (mU/L): control: 20.37 +/− 0.63, **Low dose**: **17.02 +/− 0.64 (*p* < 0.01), High dose: 13.61 +/− 1.5 (*p* < 0.0001)** T4 (ng/mL): control: 109.75 +/− 2.25, **Low dose: 96 +/− 2.75 (*p* < 0.01), High dose: 83.25 +/− 3.5 (*p* < 0.0001)** T3 (pmol/L): control: 21.30 +/− 0.51, **Low dose: 19.64 +/− 0.85 (*p* < 0.01), High dose: 15.77 +/− 0.21 (*p* < 0.0001)** Relative expression of genes in the HPT axis. Relative *Dio2* mRNA expression in the hypothalamus: control: 1.008 +/− 0.113, **Low dose: 0.121 +/− 0.008 (*p* < 0.0001), High dose: 0.0726 +/− 0.008 (*p* < 0.0001)** Relative *Mct8* mRNA expression in hypothalamus: control: 1.003 +/− 0.054, **Low dose: 0.1554 +/− 0.0156 (*p* < 0.0001), High dose: 0.1865 +/− 0.0389 (*p* < 0.0001)** Relative *Dio2* mRNA expression in pituitary: control: 1.027 +/− 0.165, **Low dose: 1.438 +/− 0.494, High dose: 5.219 +/− 2.055 (*p* < 0.001)** [**there was also a significant difference between Low dose and H-gly groups, *p* < 0.001**] Relative *Mct8* mRNA expression in pituitary: control: 0.993 +/− 0.124, **Low dose: 1.821 +/− 0.165 (*p* < 0.001), High dose: 2.793 +/− 0.207 (*p* < 0.0001)** Relative *Thrh* mRNA expression in pituitary: control: 1.005 +/− 0.078, **Low dose: 1.881 +/− 0.387 (*p* < 0.01), High dose: 3.557 +/− 0.309 (*p* < 0.0001)** Relative *Nis* mRNA expression in thyroid: **control: 1.031 +/− 0.177, Low dose: 0.5307 +/− 0.1 (*p* < 0.001), High dose: 0.1923 +/− 0.085 (*p* < 0.0001)** Relative *Tpo* mRNA expression in thyroid: control: 1.000 +/− 0.0825, **Low dose**: 0.629 +/− 0.08234, **High dose: 0.443 +/− 0.227 (*p* < 0.01)** Relative *Tg* mRNA expression in thyroid: control: 0.943 +/− 0.140, **Low dose: 0.534 +/− 0.085 (*p* < 0.001), High dose: 0.062 +/− 0.008 (*p* < 0.0001)** Relative *Tshr* mRNA expression in thyroid: control: 1.023 +/− 0.269, **Low dose: 0.185 +/− 0.054 (*p* < 0.0001), High dose: 0.054 +/− 0.008 (*p* < 0.0001)**
Docea et al. (2023) [35]	Glyphosate PRESTANA; ADI: 0.5 mg/kg body weight (bw)/day glyphosate. NOAEL: 50 mg/kg bw/day glyphosate	Female Wistar rats; Drinking water	Female rats were initially 3 months, acclimated to the laboratory environment for 2 weeks and were then mated; Exposure from GD 6 until PND 28.	Total T3 (ng/ mL). control: 39.94 ± 1.11 **ADI: 8.95 ± 0.59 (*p* < 0.001)** **NOAEL: 46.15 ± 2.83 (*p* < 0.01)** Total T4 (nmol/L). control: 13.30 ± 0.56 **ADI 19.97 ± 1.73 (*p* < 0.01)** **NOAEL 31.99 ± 1.17 (*p* < 0.001)** TSH (uUI/mL). control: 0.41 ± 0.01 **ADI: 0.42 ± 0.01 (*p* < 0.05)** NOAEL: 0.40 ± 0.01
Oliveira et al. (2023) [36]	Roundup Transorb;	Male Wistar rats; By Gavage	PND 23 at the beginning of the exposure; Exposure lasted until PND 60 (37 days of exposure) or PND 90 (67 days of exposure)	Serum TSH concentrations (ng/mL). PND 60: 0 mg GBH/kg/day: 1.00 +/− 0.14 ng/mL 0.5 mg GBH/kg/day: 1.27 +/− 0.32 ng/mL 5 mg GBH/kg/day: 1.85 +/− 0.3 ng/mL. PND 90: 0 mg GBH/kg/day: 1.45 +/− 0.43 ng/mL 0.5 mg GBH/kg/day: 1.24 +/− 0.35 ng/mL 5 mg GBH/kg/day: 0.91 +/− 0.18 ng/mL. Serum T4 concentrations (µg/dL). PND 60: 0 mg GBH/kg/day: 5.25 +/− 0.34 µg/dL 0.5 mg GBH/kg/day: 4.78 +/− 0.18 µg/dL 5 mg GBH/kg/day: 5.05 +/− 0.28 µg/dL PND 90: 0 mg GBH/kg/day: 5.05 +/− 0.28 µg/dL 0.5 mg GBH/kg/day: 5.31 +/− 0.3 µg/dL **5 mg GBH/kg/day: 5.93 +/− 0.32 µg/dL (*p* < 0.05)** Serum T3 concentrations (ng/mL) PND 60: 0 mg GBH/kg/day: 0.44 +/− 0.017 ng/mL 0.5 mg GBH/kg/day: 0.45 +/− 0.02 ng/mL 5 mg GBH/kg/day: 0.53 +/− 0.05 ng/mL PND 90: 0 mg GBH/kg/day: 0.46 +/− 0.03 ng/mL 0.5 mg GBH/kg/day: 0.46 +/− 0.04 ng/mL 5 mg GBH/kg/day: 0.49 +/− 0.05 ng/mL There were no significant differences in relative mRNA expression of *Trh* in the hypothalamus and *Trhr*, and *Tshb* in the pituitary glands. This was consistent across the different PND groups and at various concentrations of GBH.
Elkattan et al. (2024) [28]	Glyphosate; 1 mL glyphosate solution 24%	Adult male albino rats; Daily, orally	Adult rats; Exposed for 3 weeks	Free serum T3 levels (ng/dL). Week 1: control: 4.853 +/− 0.147, Gly: 3.637 +/− 0.364 Week 2: control 5.059 +/− 0.235, **Gly 3.02 +/− 0.215 (*p* < 0.05)** Week 3: control 5.183 +/− 0.201, Gly 3.118 +/− 0.245 Free serum T4 levels (pg/mL). Week 1: control 2.686 +/− 0.41, **Gly 1.776 +/− 0.188 (*p* < 0.05)** Week 2: control 3.018 +/− 0.189, **Gly 1.46 +/− 0.329 (*p* < 0.05)** Week 3: control 3.109 +/− 0.137, **Gly 1.542 +/− 0.436 (*p* < 0.05)** Total T3 (ng/dL). Week 1: control 126.9 +/− 7.2, **Gly 93.9 +/− 13.1 (*p* < 0.05)** Week 2: control 125.3 +/− 8.9, **Gly 80.56 +/− 3.88 (*p* < 0.05)** Week 3: control 129.7 +/− 8.3, **Gly 86.4 +/− 0.82(*p* < 0.05)** Total T4 (µg/dL). Week 1: control 7.789 +/− 1.024, **Gly 3.935 +/− 0.667 (*p* < 0.05)** Week 2: control 7.268 +/− 0.862, **Gly 3.789 +/− 0.683 (*p* < 0.05)** Week 3: control 7.333 +/− 0.927, **Gly 4.325 +/− 0.504 (*p* < 0.05)** TSH (μU/mL). Week 1: control 0.0144 +/− 0.0098, Gly 0.00862 +/− 0.00123. Week 2: control 0.0128 +/− 0.0065, Gly 0.00903 +/− 0.0013. Week 3: control 0.0226 +/− 0.0065, **Gly 0.199 +/− 0.0004 (*p* < 0.05)**

Bolded values are used to indicate statistical significance. OR = Odds Ratio; HR = Hazard Ratio; CI = Confidence Interval; GD = Gestational Day; PND = Postnatal Day; bw = Body Weight; ADI = Acceptable Daily Intake; NOAEL = No Observed Adverse Effect Level; Thrα1/Thrβ1 = Thyroid Hormone Receptor alpha 1/beta 1; Dio2/Dio3 = Type II/III Deiodinase; Mct8 = Monocarboxylate Transporter 8; Oatp1c1/Slco1c = Organic Anion Transporting Polypeptide 1c1; Tg = Thyroglobulin; Tpo = Thyroid Peroxidase; Tshr = Thyroid-Stimulating Hormone Receptor; Nis = Sodium/Iodide Symporter.

**Table 5:** Summary of other animal model studies investigating the impact of glyphosate exposure on thyroid-related outcomes (hormone levels/gland effects).

Author (Year)	Chemical Exposure and Dose	Experimental Model and Exposure Route	Age at the Beginning of the Exposure and Exposure Duration	Association with Thyroid Disease
Liu et al. (2022) [37]	Glyphosate; 0.7, 7, and 35 mL/L of glyphosate	Embryo-larval zebrafish (*Danio rerio*); Water	1–1.5 h post-fertilization; Exposure from 3 to 120 h post-fertilization	Ratio of T3 to T4 Control 0.0209 +/− 0.0013 0.7 mg/L gly 0.0167 +/− 0.0029 7 mg/L gly 0.0196 +/− 0.0026 **35 mg/L gly 0.0148 +/− 0.0009 (*p* < 0.05)** T3: Control: 9.31 +/− 0.65 pmol/L 0.7 mg/L gly: 8.45 +/− 1.29 pmol/L 7 mg/L: 8.52 +/− 1.08 pmol/L, **35 mg/L: 7.61 +/− 0.37 pmol/L (*p* < 0.05)** T4: Control: 441.7 +/− 7.9 pmol/L **0.7 mg/L gly: 504.4 +/− 9.6 pmol/L (*p* < 0.01)** 7 mg/L: 435.5 +/− 9.2 pmol/L **35 mg/L: 513.6 +/− 25.4 pmol/L (*p* < 0.01)**
Vardakas et al. (2022) [38]	Glyphosate and Roundup; 5 mg/kg/day	New Zealand rabbits; Dissolved in a 5% ethanol/water solution and administered once daily	2–3 months old; Exposed for 12 months	Thyroid gland GSH (reduced form of glutathione) concentrations (µmol/mg protein). Control 0.0075 +/− 0.0018 Glyphosate 0.0096 +/− 0.0018 Roundup 0.0132 +/− 0.0044 Thyroid gland catalase activity (U/mg protein). Control 67.84 +/− 7.36 Glyphosate 62.29 +/− 6.43 Roundup 48.78 +/− 6.61 Thyroid gland total antioxidant capacity (mmol DPPH/mg protein). Control 0.0681 +/− 0.0096 Glyphosate 0.0764 +/− 0.01 Roundup 0.0747 +/− 0.0066 Thyroid gland thiobarbituric acid reactive substances (nmol/mg protein). Control 1.37 +/− 0.06 Glyphosate 1.15 +/− 0.11 Roundup 0.99 +/− 0.05 Thyroid gland protein carbonyls concentration (nmol/mg protein) Control 1.51 +/− 0.44 Glyphosate 0.952 +/− 0.198 Roundup 1.28 +/− 0.4
Killian et al. (2023) [39]	Roundup; 0.05, 0.5, 5, 10, and 20 mg/L of glyphosate	Japanese medaka larvae (Oryzias latipes); Well plate containing exposure solution	8 h post fertilization; Exposure from 8 h post-fertilization to 14 days post-fertilization	Relative expression of thyroid hormone receptor alpha. Control (0 mg/L) 1.523 +/− 0.746 **0.05 mg/L gly 0.274 +/− 0.182 (*p* < 0.05)** 0.5 mg/L gly 1.53 +/− 0.909 5 mg/L gly 0.544 +/− 0.233 0 mg/L gly 0.989 +/− 0.419 **20 mg/L gly 0.306 +/− 0.17 (*p* < 0.05)** Relative expression of thyroid hormone receptor beta. Control (0 mg/L) 1.22 +/− 0.437 **0.05 mg/L gly 0.426 +/− 0.181 (*p* < 0.05)** 0.5 mg/L gly 2.20 +/− 1.045 5 mg/L gly 0.757 +/− 0.379 10 mg/L gly 1.45 +/− 0.59 **20 mg/L gly 0.264 +/− 0.132 (*p* < 0.05)** Relative expression of thyroid-stimulating hormone beta. Control (0 mg/L) 1.44 +/− 0.55 **0.05 mg/L gly 1.59 +/− 0.77 (*p* < 0.05)** 0.5 mg/L gly 6.30 +/− 3.89 5 mg/L gly 1.09 +/− 0.82 10 mg/L gly 2.55 +/− 1.01 20 mg/L gly 0.290 +/− 0.155

Bolded values are used to indicate statistical significance. T3 = Triiodothyronine; T4 = Thyroxine; TRα/TRβ = Thyroid Hormone Receptors alpha/beta; TSHβ = Thyroid-Stimulating Hormone beta; GSH = Reduced Glutathione; DPPH = 2,2-diphenyl−1-picrylhydrazyl (total antioxidant capacity assay); U/mg protein = Units per milligram of protein; µmol/mg protein = Micromole per milligram protein; nmol/mg protein = Nanomole per milligram protein.

## Data Availability

No new data were created or analyzed in this study. Data sharing is not applicable to this article.

## Abbreviations

The following abbreviations are used in this manuscript:

AHS	Agricultural Health Study
AMPA	Aminomethylphosphonic acid
Bw	Body weight
CI	Confidence Interval
EDC	Endocrine-disrupting chemical
FAE	Formulation acid equivalent
FRTL−5	Fisher Rat Thyroid Cell Line−5
FT3	Free triiodothyronine
FT4	Free thyroxine
GD	Gestational day
GBH	Glyphosate-based herbicide
GS	Gosner stage
HR	Hazard stage
HPT	Hypothalamic–pituitary–thyroid (axis)
mRNA	Messenger Ribonucleic acid
NOAEL	No observed adverse effect level
OR	Odds ratio
PND	Postnatal day
RT-PCR	Reverse transcription polymerase chain reaction
SE	Standard error
T3	Triiodothyronine
T4	Thyroxine
TG	Thyroglobulin
Thrα1	Thyroid hormone receptor alpha 1
Thrβ1	Thyroid hormone receptor beta 1
Thrβ2	Thyroid hormone receptor beta 2
TRH	Thyrotropin-releasing hormone
TSH	Thyroid-stimulating hormone
TSHR	Thyroid-stimulating hormone receptor
TTF−1	Thyroid transcription factor 1
VM	VisionMax^®^
